# Bioactive Collagen Hydrolysate-Chitosan/Essential Oil Electrospun Nanofibers Designed for Medical Wound Dressings

**DOI:** 10.3390/pharmaceutics13111939

**Published:** 2021-11-16

**Authors:** Maria Râpă, Carmen Gaidau, Liliana Mititelu-Tartau, Mariana-Daniela Berechet, Andrei Constantin Berbecaru, Irina Rosca, Aurica P. Chiriac, Ecaterina Matei, Andra-Mihaela Predescu, Cristian Predescu

**Affiliations:** 1 Faculty of Materials Science and Engineering, University Politehnica of Bucharest, 313 Splaiul Independentei, 060042 Bucharest, Romania; rapa_m2002@yahoo.com (M.R.); andrei_berbecaru@yahoo.com (A.C.B.); ecaterinamatei@gmail.com (E.M.); andrapredescu@yahoo.com (A.-M.P.); cpredescu56@yahoo.com (C.P.); 2 The National Research & Development Institute for Textiles and Leather—Division Leather and Footwear Research Institute Bucharest, 93 Ion Minulescu Street, 031215 Bucharest, Romania; marianadanielaberechet@yahoo.co.uk; 3 Pharmacology, Clinical Pharmacology and Algesiology Department, Faculty of Medicine “Grigore T. Popa”, University of Medicine and Pharmacy, 16 Universitatii, 700115 Iasi, Romania; lylytartau@yahoo.com; 4 Centre of Advanced Research in Bionanoconjugates and Biopolymers Petru Poni, Institute of Macromolecular Chemistry, 41A Aleea Grigore Ghica-Voda, 700487 Iasi, Romania; rosca.irina@icmpp.ro; 5 Department of Natural Polymers, Bioactive and Biocompatible Materials, Petru Poni Institute of Macromolecular Chemistry, 41 A Grigore Ghica Voda Alley, 700487 Iasi, Romania; achiriac@icmpp.ro

**Keywords:** wound dressing, collagen hydrolysate and chitosan, lemon balm essential oil, dill essential oil, in vivo biocompatibility, antimicrobial activity

## Abstract

In this study, lemon balm (*Melissa officinalis* L.) and dill (*Anethum graveolens* L.) essential oils (EOs) were encapsulated into collagen hydrolysates extracted from bovine tendons and rabbit skins, both mixed with chitosan (CS) by using the coaxial electrospinning technique for potential wound dressing applications. The morphology and chemical composition of the electrospun nanofibers were investigated using scanning electron microscopy (SEM) and attenuated total reflectance Fourier transform infrared spectroscopy (ATR-FTIR). The antimicrobial activity of the dill EO and lemon EO, as well as the electrospun samples loaded with essential oils was determined by disk diffusion assay against *Staphylococcus aureus* ATCC 25923, *Escherichia coli* ATCC 25922, *Enterococcus faecalis* ATCC 29212, and *Salmonella typhimurium* ATCC 14028 bacterial strains; *Candida albicans* ATCC 10231 and *Candida glabrata* ATCC 90028 yeast strains; and *Aspergillus brasiliensis* ATCC 9642 fungal strain. In vivo biocompatibility testing of the collagen hydrolysate-chitosan/essential oil electrospun nanofibers was based on the determination of the hematological, biochemical, and immunological profile and the evaluation of the influence produced on the oxidative stress in white Swiss mice. The synergetic effect of dill and lemon balm EOs can improve the antimicrobial activity of collagen hydrolysate-chitosan nanofibers against the most important bacterial strains. The in vivo test results suggested a good biocompatibility of electrospun samples based on collagen hydrolysate extracted from bovine tendons or rabbit skin mixed with chitosan and containing dill and/or lemon balm essential oils as encapsulated bioactive compounds.

## 1. Introduction

Wound dressing materials are produced for wound healing process. The main techniques to obtain wound dressings include electrospinning [1,2,3,4,5,6,7,8,9], cryogelation [10], solvent casting [11], freezing-thawing [12,13], and dip coating [14] methods. Usually, wound dressings include films, bandages, hydrocolloids, fibers, foams, dermal patches, and sponges [15]. The electrospinning process can be used to fabricate porous nanofibers and introduce the desired components to provide bioactive properties to wound dressings [16]. For example, beta-estradiol, a therapeutic agent, was introduced to a polyurethane-dextran composite nanofibrous wound dressing [3]. In another paper [7], gentamicin was loaded into by-layer scaffold based on polyvinylpyrrolidone gelatin and cellulose acetate. The disadvantage of these traditional antimicrobial agents is related to antibiotic resistance. The alternative to topical antimicrobial agents is to develop new wound healing materials based on alginate [17], chitosan [6,18], collagen [19], nanocellulose [20], inorganic antimicrobial agents such as zinc oxide [5,21], and plant extracts [22].

The application of animal-derived proteins in electrospinning for wound dressings as compared with synthetic polymers is an environmentally friendly approach because the non-toxic solvents are used for preparing solutions, and, in addition, they possess antimicrobial and biocompatibility properties [19,23]. Collagen is the most abundant protein in mammals, being a major constituent of skin, bones, tendons, blood vessels, and heart tissue, and successfully used for in vitro and in vivo tissue regeneration engineering [24,25,26,27,28].

Chitosan is a cationic polysaccharide obtained from crustaceans with outstanding biocompatibility, biodegradability, antimicrobial, and antifungal activities [29]. It is a very suitable biopolymer for the release of drugs in the treatment of diseases and wound healing [30]. The structure of chitosan contains active hydroxyl and amino groups with hydrogen bonds between its chains. Chitosan nanofibers can be obtained by the electrospinning method using acetic acid (90%), trifluoroacetic acid, and solvents/co-solvents. The electrospinnability of the pure chitosan solution is weak due to its high viscosity, and intensity of inter- and intracatenary hydrogen bonds. To eliminate these problems, before electrospinning, chitosan solutions are mixed either with various synthetic polymers, metal nanoparticles, nanoclays, mineral compounds, metal oxides, gold, silver, clays, zeolites, or organic metal structures [31]. The mixing of chitosan with collagen leads to chitosan-collagen complexes, which are polyelectrolytes showing excellent physical and chemical properties useful for biological field [32]. In addition, these complexes increase the biocompatibility, drug delivery capacity, and mechanical strength of chitosan nanofibers [30].

Plant extracts, in particular essential oils (EOs), constitute a promising replacement to synthetic drugs, due to their antibacterial, antifungal, anti-inflammatory, and antioxidant activities [16,33]. The main drawbacks of essential oils are related to their volatility and degradation under processing temperature. Therefore, to avoid these deficiencies in wound care treatment, the incorporation or encapsulation of EO into polymeric matrices by electrospinning could be a good strategy [31,34]. In addition, polyphenols from EOs can cross-link collagen, via hydrogen bonding, for an increase in their mechanical strength and thermal stability [35]. Thus, *Gastrodia elata* and tea tree oil have been incorporated into silk fibroin protein to fabricate all-natural foam dressings with anti-inflammatory reduction in the formation of nitrite as compared with the untreated group [34]. In another study, the clove essential oil incorporated into electrospun zein/polyethylene oxide (PEO)/fibrous meshes showed good antibacterial activity against both *S. aureus* and *E. coli* after 24 h of incubation and good biocompatibility promoting wound healing of mice skin wound models after 11 days [8]. Liakos et al. [9] incorporated up to 25% *v*/*v* of cinnamon or lemongrass or peppermint EO to a solution of cellulose acetate and reported antimicrobial activity towards *E. coli*, and a good biocompatibility on skin cells model. Nanofibers based on concentrated collagen hydrolysate loaded with thyme and oregano EOs demonstrated antimicrobial activity against *S. aureus*, *E. coli*, *P. aeruginosa* and *C. albicans* and in vitro biocompatibility with NCTC clone 929 of fibroblastic cell [36]. The significant inhibition of Gram negative (*Escherichia coli* S17) and Gram positive (*S. aureus* ATCC 25923) bacteria growth was reported for polycaprolactone (PCL)/polyvinyl acetate (PVAc) asymmetric membranes loaded with thyme EO rich in bactericidal monoterpene carvacrol (CRV) [37]. Recently, *Hypericum perforatum* oil prepared by the electrospinning method was studied on an experimental diabetic wound model to compare the diabetic wound healing effect [38].

In this study, the singular effect of dill EO and lemon balm EO, and their dual contributions to the bioactive properties of the electrospun collagen hydrolysate with chitosan were investigated. Dill (*Anethum graveolens* L.) essential oil is known for its remarkable anti-inflammatory and analgesic properties, superior to Diclofenac [39] and lower antimicrobial activity as compared with other essential oils [39,40]. Instead, lemon balm (*Melissa officinalis* L.) essential oil is recognized for its antimicrobial activity against nosocomial infections [40,41]. To the best of our knowledge, nanofibers containing encapsulated dill EO and lemon balm EO into collagen-chitosan complexes have not been reported. The reason for this choice was justified by their anti-inflammatory and antimicrobial effect and the much safer alternative for wound healing management as compared with synthetic compounds. The combination of these essential oils encapsulated into natural polymers, such as collagen extracted from beef tendons and rabbit skins, and mixed with chitosan is expected to provide a synergistic activity, proven by increasing the area of inhibition in microbiological tests. A comprehensive evaluation of the antimicrobial activity and in vivo biocompatibility are provided.

## 2. Materials and Methods

### 2.1. Materials

The collagen hydrolysate extracted from bovine tendons (HCB) was obtained by enzymatic process [23]. The collagen hydrolysate extracted from rabbit collagen glue (HCR) was prepared from preliminarily crushed pickled rabbit skin by boiling in a water bath at a temperature of 90 °C, for four hours, according to our previous studies [21,23]. Chitosan (CS) derived from crab shells is highly viscous (Sigma, Riedst, Steihtein, Germany) and, in the form of crystals, is characterized by a viscosity (1% in acetic acid, at 20 °C) of 1267 MPa·s and a sulphated ash content of 0.2%. Lemon balm (L) (*Melissa officinalis* L.) and dill (D) (*Anethum graveolens* L.) essential oils were acquired from SOLARIS PLANT SRL, Bucharest, Romania. The main constituents of the essential oil of *M. officinalis* are citrals (geranial and neral, 9.9%), citronellal (13.7%), limonene (2.2%), geraniol (3.4%), *β-*caryophyllene (4.6%), *β-*caryophyllene oxide (1.7%), and germacrene D (2.4%) [42]; in the case of dill EO, the main constituents are *o*-cimol (30.71%) and α-felandren (23.21%) [43]. Table 1 shows the total phenolic content (TPC) and the antioxidant activity for EOs at a concentration in ethanol of 60 mg mL^−1^ determined by using the DPPH (2,2-diphenyl-1-picrylhydrazyl) and 2,2′-azino-bis (3-ethylbenzthiazoline)-6-sulfonic acid (ABTS^+^^·^) methods.

### 2.2. Methods

#### 2.2.1. Collagen Hydrolysate’s Characterization

The collagen hydrolysates in powder form were analyzed according to the standards for volatile matters (SR EN ISO 4684:2006), ash content (SR EN ISO 4047:2002), total nitrogen and protein content (SR EN ISO 5397:1996), conductivity (SR EN 2788:1997), pH (STAS 8619/3:1990), and in house methods (aminic nitrogen and molecular weight). The average particle size, polydispersity, and zeta potential of collagen hydrolysates in 12% aqueous solutions were determined by Zetasizer Nano-ZS (Malvern, Malvern Hills, UK). The analyses were performed in triplicate and the results were expressed as the average values.

#### 2.2.2. Preparation of Electrospinning Solutions

The 2.66% (*w*/*v*) solutions of HCB and HCR, respectively, were prepared by gentle mixing of each HCB and HCR solid extract with 1.5% (*w*/*v*) solution of CS previously prepared in acetic acid at a concentration of 80% (*v*/*v*) under magnetic stirring at 800 rpm, and 90 °C for 3 h. The prepared solutions were kept for 30 min in ultrasonic equipment for obtaining homogeneous solutions. The HCB-CS and HCR-CS solutions showed a pH (at 24.1 °C) of 2.5 (Consort C831 Multiparameter analyzer, Turnhout, Belgium) and the electrical conductivity (at 25 °C) (C1010, Consort Turnhout, Belgium) of 1236 µS/cm and 1316 µS/cm, respectively.

Before encapsulation into HCB and HCR solutions, each dill and lemon balm EO was dispersed into ethanol to reach a concentration of 60 mg mL^−1^.

#### 2.2.3. Encapsulation of Lemon Balm and Dill Essential Oils

A commercial TL-Pro-BM Electrospinning equipment (Tong Li Tech Co., Ltd., Bao An, Shenzhen, China), which included a dual syringe pump, a high-voltage power supplier, a coaxial stainless steel needle, and a coated aluminum foil collector coupled to a ground electrode was employed for encapsulation of dill and lemon balm essential oils into hydrolyzed collagen/chitosan solutions.

Table 2 shows the compositions and codes for the prepared electrospinning nanofiber samples. Electrospun HCB-CS and HCR-CS nanofibers were provided as controls. A mixture of dill essential oil/lemon balm essential oil (1:1) was also encapsulated into each HCB-CS and HCR-CS solution, to assess their synergic effect.

Twenty mL of HCB-CS and HCR-CS solutions, respectively, were loaded into a 25 mL plastic syringe. Each HCB-CS and HCR-CS solution was forced to pass from the syringe into the outer inlet of the coaxial stainless steel needle, through silicon tubing at a flow rate of 1 mL/h and a voltage in the range of 21–28 kV. In the coaxial electrospinning technique, the EO agents were introduced into another syringe (core solution) and perfused into the inner needle of the coaxial stainless steel needle, at a flow rate of 0.3 mL/h. Theoretically, the volume ratio of polymeric solution to EO solution is 1:0.3. For the HCB-CS experiments the distance from the coaxial stainless steel needle to the collector was 10 cm, while in the case of HCR-CS, the distance to produce nanofibers was 5 cm. This correlated with the higher viscosity of HCR as compared with that of the HCB solution. The aluminum surfaces with dimensions of (10 × 20) cm^2^ were coated with nanofibers during a deposition time of 30 min. All experiments were performed at a temperature of 21.9 ± 0.2 °C, and a relative humidity of 29%.

#### 2.2.4. EO Loading Efficiency

The quantity of EO encapsulated onto the collagen-chitosan nanofibers was estimated via UV-Vis spectroscopy using a UV-Vis spectrophotometer. Predetermined EO-specific calibration curves (0–60 mg mL^−1^) in ethanol were performed. The nanofibers containing encapsulated EOs were immersed into ethanol and mixed at room temperature for 24 h. The supernatant was filtered (0.2 µm membrane filter) and the absorbance value analyzed at 235 nm. The Equation 1 used to measure the loading efficiency was the following:

(1) $$Loading\;Efficiency\;(\%)=\frac{EO\;measured\;amount}{EO\;theoretical\;amount}\times 100$$

The theoretical amount of EO was 23%.

#### 2.2.5. Scanning Electron Microscopy (SEM)

The analyses for nanofiber morphology and size distribution were performed using a SEM (FEI Quanta 200 Scanning Electron Microscope, Eindhoven, The Netherlands). The electrospun samples were coated with a thin Au layer of about 5 nm, in order to avoid charging effects. The average fiber diameters were determined using OriginPro 7.5 (OriginLab, Northampton, MA, USA) by processing the manual measurement of 20 identified fibers.

#### 2.2.6. Attenuated Total Reflectance Fourier Transform Infrared Spectroscopy (ATR-FTIR)

The FTIR investigation of dill EO, lemon balm EO, HCB-CS nanostructures, HCR-CS nanostructures, and encapsulated EOs into nanofibers was performed using an INTERSPEC 200-X spectrophotometer (Interspectrum, Tartumaa, Estonia) ranging from 700 to 4000 cm^−1^ having 20 scans with resolution at 2 cm^−1^. The EOs samples were placed on a Zn-Se ATR crystal with the help of a Pasteur pipette. The chitosan was assessed as film obtained during the preparation step for the electrospinning process. The attenuate total reflectance (ATR) crystal was cleaned with ethanol prior to each spectral acquisition.

#### 2.2.7. Antimicrobial Activity

The antimicrobial activities of the dill EO and lemon EO, as well as of the electrospun samples loaded with essential oils, were determined by disk diffusion assay [44] against seven different reference strains: bacterial strains represented by *Staphylococcus aureus* ATCC 25923, *Escherichia coli* ATCC 25922, *Enterococcus faecalis* ATCC 29212, and *Salmonella typhimurium* ATCC 14028; yeast strains represented by *Candida albicans* ATCC 10231 and *Candida glabrata* ATCC 90028; and the fungal strain *Aspergillus brasiliensis* ATCC 9642.

All microorganisms were stored at −80 °C in 20–40% glycerol. The bacterial strains were refreshed in tryptic soy broth (TSB) and nutrient broth (NB) at 36 ± 1 °C. The yeast and fungal strains were refreshed on Sabouraud dextrose broth (SDB) and potato dextrose broth (PDB), respectively, at 25 ± 1 °C. Microbial suspensions were prepared with these cultures in sterile solution to obtain turbidity optically comparable to that of 0.5 McFarland standards. Volumes of 0.2 mL from each inoculum were spread on the Petri dishes. The sterilized paper disks (6 mm) were placed on the plates and an aliquot (50 μL) of the samples was added. To evaluate the antimicrobial properties, the growth inhibition was measured under standard conditions after 24 h of incubation at 36 ± 1 °C for the bacterial and the yeast strains and after 48 h at 25 ± 1 °C for the fungal strain. All tests were carried out in triplicate to verify the results. After incubation, the diameters of inhibition zones were measured by using Image J version 1.52 t software (National Institutes of Health, Bethesda, MD, USA) [45].

All data were expressed as the mean ± standard deviation of the mean. Statistical analysis was performed with XLSTAT Ecology version 2019.4.1 software (Addinsoft, New York, NY, USA).

#### 2.2.8. In Vivo Biocompatibility 

**Figure 1:**
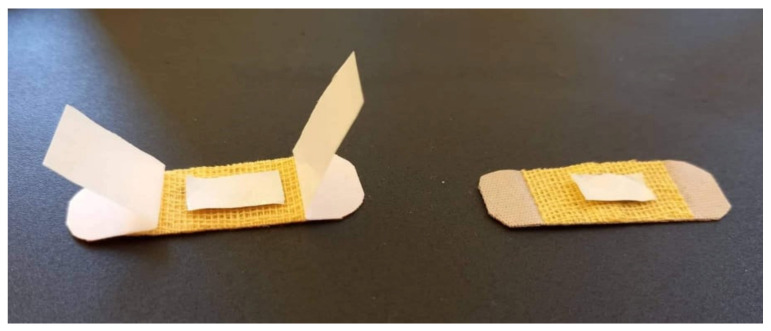
Illustration of preparing nanofiber samples prior to being applied to the animal group.

In the experiment on biocompatibility testing, white Swiss adult mice were used (weighing between 25 and 30 g, 3 months old), with uniform sex distribution, from the Cantacuzino Institute Bucharest, Baneasa Resort, through the biobase of “Grigore T. Popa” University of Medicine and Pharmacy from Iaşi. The animals were brought a week before, for accommodation, kept in standard laboratory conditions (with a constant temperature of 21 ± 2 °C, relative humidity of 50–70%, and alternating lighting regime (light/dark ratio = 12 h/12 h), in individual cages, with food and water available ad libitum. To avoid chronobiological influences, the tests were performed in the interval between 8 and 12 a.m. Throughout the study, the recommendations of the University Ethics Commission were followed, in strict accordance with the international ethical regulations, regarding work on laboratory animals. On the first day of the experiment, the animals were anesthetized, using ketamine 50 mg/kg, and xylazine 10 mg/kg, intraperitoneally administered. Subsequently, the skin in the left dorsal region was shaved and a superficial incision 1 cm long was made, parallel to the spine. The nanofiber samples with dimensions of 1 × 0.5 cm were positioned on the sterile textile material of a patch and applied directly over the incision area, fixing it on the skin using an adhesive system (Figure 1). A dry patch with sterile textile material was applied to the animals in the control group.

Randomly, 9 batches of 5 mice each received the nanofiber test samples.

Throughout the experiment, the behavior of the animals was observed (spontaneous motility, food and water consumption, and stereotypical movements), and on the 7th day the macroscopic aspect of the incision area was evaluated to observe local tissue changes.

In vivo testing of the biocompatibility of the studied substances was based on the assessing of the white blood count of the hematological, biochemical, and immunological profile and on the evaluation of the influence on the oxidative stress in the animals that received the tested electrospun samples. At 24 h and 7 days after the application of the electrospun samples, the animals were anesthetized with 1% isoflurane and blood was collected from the lateral vein of the tail, to evaluate: the percentage of components in the leukocyte formula, the glutamic-pyruvic transaminase (TGP), glutamic-oxaloacetic transaminase (TGO), and lactate dehydrogenase (LDH), as well as the serum levels of urea and creatinine [46,47]. In order to easily collect blood samples, the tail of the animal was placed in warm water (at 40 °C) to dilate the lateral vein. The tail was kept in a stretched position, the lateral caudal vein was identified, at a distance of 3 cm from the tip, and the respective area was antisepticised with 70% alcohol [48,49,50]. Under local anesthesia with 1% benzocaine (sprays), the vein was punctured and a blood sample was taken [51]. To assess the hemoleucogram, 0.3 mL of venous blood was collected in vacutainers containing EDTA as anticoagulant tripotassium/dipotasium/disodium (vacutainer with purple/pink cap, K3 EDTA). The device used was HEMAVET 950 (Oxford, UK), an automatic analyzer working on the principle of fluorescence flow cytometry.

For biochemical determinations, 0.3 mL of venous blood was collected on an empty stomach, on heparin, and the samples were analyzed using the ACCENT 200 biochemistry analyzer (Cormay, Warsaw, Poland).

To evaluate the influence on oxidative stress, the following specific parameters were evaluated: superoxide dismutase (SOD) and glutathione peroxidase (GPx). Determination of serum SOD activity was performed by colorimetric method with xanthine and xanthine oxidase, using a RANSOD kit from RANDOX Laboratories Ltd. (Warsaw, Poland) on blood samples (0.3 mL) collected on heparin. To determine GPx, 0.3 mL of blood was collected in heparinized vacutainers, and the activity of this enzyme was evaluated by enzymatic method, using a RANSEL kit from RANDOX Laboratories Ltd. (Warsaw, Poland).

After 7 days in the experiment, serum opsonic capacity (OC) was measured (using cultures of *Staphylococcus aureus* 94). At the end of the test, the animals were sacrificed under general anesthesia with 2% isoflurane [46,47], and the peritoneal macrophages were removed from the intact peritoneal cavity by washing with 10 mL HANKS solution (thermostated at 37 °C). The samples were centrifuged (1000 rotations per minute, 10 min), placed in contact with *Staphylococcus aureus* 94 cultures, incubated for 48 h at 37 °C, and reseeded on culture media. The following immune parameters were evaluated: phagocytic capacity (PC) and bactericidal capacity (BC) of peritoneal macrophages [52].

Euthanasia was performed without physical and mental suffering, with rapid onset of unconsciousness, cardiac arrest, stopping breathing, and death. This is a standard procedure and has been performed in special autopsy rooms, separate from the place where other animals are [53,54]. The results obtained were expressed as the arithmetic mean ± standard deviation (SD) of the mean values for each assessed parameter and for each studied substance and were statistically processed using the SPSS program version 17.0 (Armonk, NY, USA) for Windows 10 and the one-way ANOVA method. These made it possible to assess the significance of the differences recorded in the same group of animals, as well as the differences found between the groups, i.e., those that received the nanofiber-based patches with bioactive substances as compared with the control group. Values of the *p* coefficient (probability) lower than 0.05 were considered to be statistically significant.

## 3. Results and Discussion

### 3.1. Physical-Chemical Characteristics of HCB and HCR Extracts

The collagen hydrolysate characteristics presented in Table 3 show high protein content with different molecular weights, in agreement with aminic nitrogen content; bovine collagen had higher molecular weight and lower aminic concentration. The high difference in electric conductivity can explain the more structured nanofibers made with rabbit collagen hydrolysate due to the higher conductivity. We attributed the difference of electric conductivity to the slightly higher content in salts and associative properties of collagen particles (1.61% ash content and 926.7 nm average particle size).

### 3.2. Efficiency of Essential Oils Encapsulation

**Figure 2:**
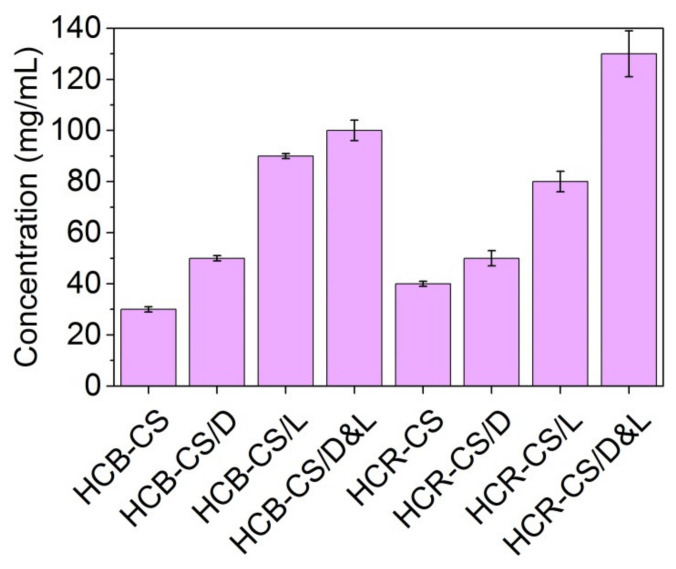
The concentration of EOs found in collagen hydrolysate-chitosan/essential oils electrospun nanofibers.

It was found that the amounts of dill EO and lemon balm EO encapsulated into collagen hydrolysate-chitosan nanofibers were in the range of 50 ± 1.2 mg mL^−1^ and 130 ± 9.1 mg mL^−1^, respectively (Figure 2). The loading efficiencies as calculated with Equation (1) were in the range from 21.7% to 56.5%, the higher values being obtained for nanofibers containing both dill and lemon balm EOs. The high values obtained in the case of encapsulated lemon balm EO can be explained by their flash points. The flash point for *Melissa officinalis* essential oil is 85 °C, while for dill essential oil, the flash point is 48 °C. A similar result (29–39% efficiency to encapsulation) was reported in the case of thyme essential oil and oregano essential oil loaded into collagen [36].

### 3.3. Scanning Electron Microscopy (SEM) Analysis

**Figure 3:**
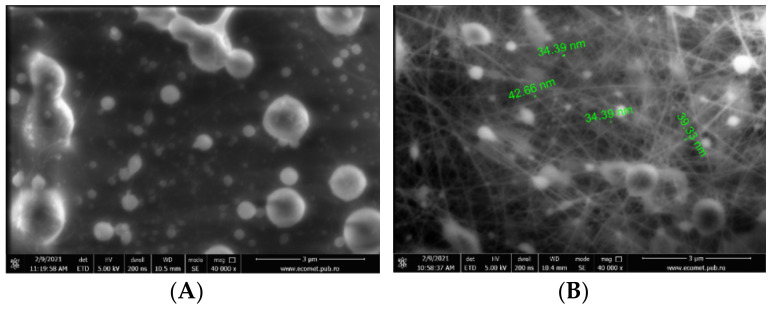

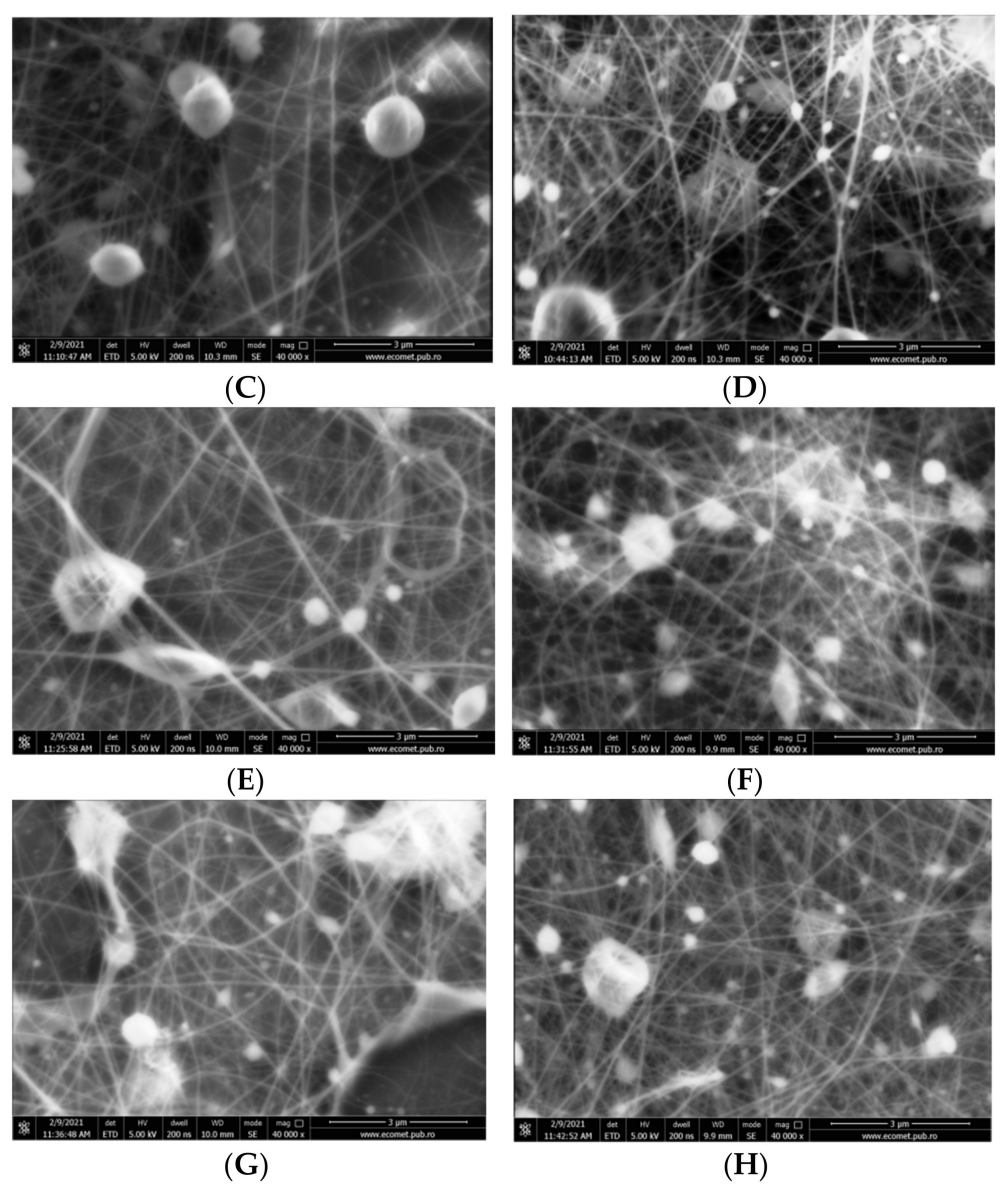
HCB-CS nanofibers (**A**); HCB-CS/Dill EO nanofibers (**B**); HCB-CS/ Lemon balm EO nanofibers (**C**); HCB-CS/Dill EO&Lemon balm EO nanofibers (**D**); HCR-CS nanofibers (**E**); HCR-CS/Dill EO nanofibers (**F**); HCR-CS/Lemon balm EO nanofibers (**G**); HCR-CS/Dill EO&Lemon balm EO nanofibers (**H**).

Spherical shapes of collagen hydrolysate-chitosan nanofibers with micrometric dimension were observed in Figure 3A, B due to the interaction between components. Encapsulation of EOs into collagen-chitosan matrix reduced the number of spherical particles facilitating more interactions between components with the beneficial contribution to bioactive wound dressing application. Such surface morphology of chitosan-collagen with spherical structure was also observed by Hua et al. [32].

Nanofibers loaded with EOs showed from 471 to 580 nm dimension sizes [36].

**Figure 4:**
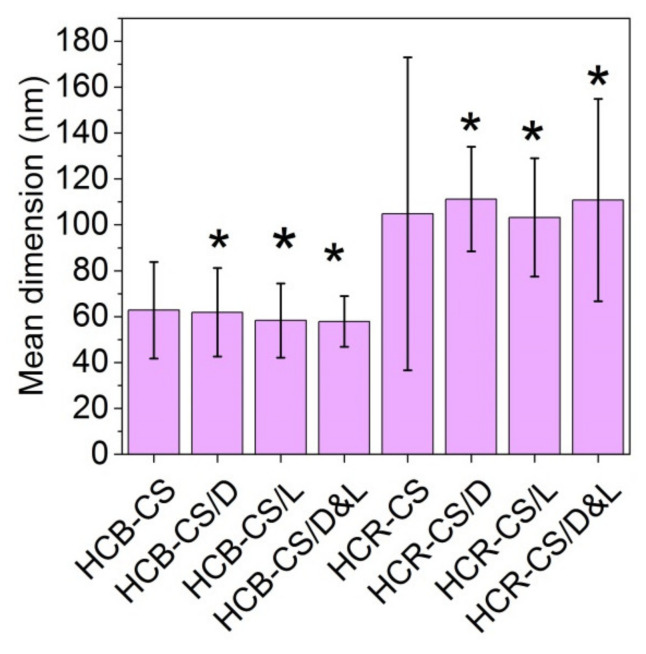
Average size nanofibers samples. Significant differences between the means of dimensions and the control (HCB-CS and HCR-CS) for * *p* < 0.05.

From Figure 4, it can be noticed that the thinner nanofibers of 60 nm were made from bovine collagen hydrolysate as compared with rabbit collagen nanofibers, with an average size around 120 nm.

### 3.4. Attenuated Total Reflectance Fourier Transform Infrared Spectroscopy (*ATR*-*FTIR*) Analysis

**Figure 5:**
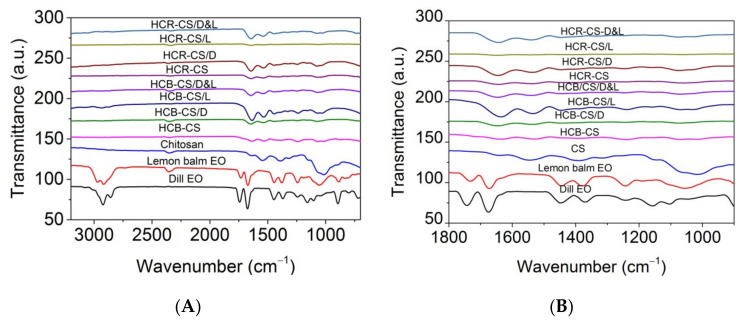
ATR-FTIR spectra for collagen hydrolysate-chitosan/essential oils electrospun nanofibers as compared with dill EO, lemon balm EO, and chitosan in the range between 4000 and 700 cm^−1^ (**A**); between 1800 and 900 cm^−1^ (**B**).

FTIR spectra of essential oils, chitosan, and loaded essential oils into HCB-chitosan and HCR-chitosan, respectively, are shown in Figure 5A,B.

Dill EO showed characteristic peaks at 1045 cm^−1^, 1104 cm^−1^ (–C–O–C stretching), 1447 cm^−1^ (C=C bending), 2858 cm^−1^ (–CH stretching at methylene hydrogen), and 2923 cm^−1^ (asymmetric –CH stretching), similar with those reported by Das et al. [55], i.e., 1742 cm^−1^, 1675 cm^−1^ (C=O stretching vibrations), 1339 cm^−1^ (C–H bending vibrations of alkanes), 1242 cm^−1^, 1156 cm^−1^ (OH bending vibrations of phenols), 1045 cm^−1^, 963 cm^−1^, 894 cm^−1^ (C–H stretching vibrations of aromatics), and 801 cm^−1^ (C=C bending vibrations of alkanes). The ATR-FTIR spectrum for chitosan shows the following absorption bands: 3261 cm^−1^ (–NH stretching), 2985 cm^−1^ (C–H stretching), 1639 cm^−1^ (bending vibration of –NH_2_ groups), 1545 cm^−1^ (*N*-acetyl group content), 1319 cm^−1^ and 1017 cm^−1^ (O–H group stretching vibrations) [21].

The spectra of HCB-CS and HCR-CS complexes indicated that the amide I of chitosan from 1639 cm^−1^ (stretching vibrations of peptide C=O groups) moved to 1663 cm^−1^ and 1635 cm^−1^, respectively. The amide II (1545 cm^−1^) associated with the secondary structure in chitosan was not found in collagen-chitosan complexes and encapsulated EOs. This indicated that the –NH_2_ and –OH groups in the chitosan chain participate in the reaction [32]. The N–H bending vibrations coupled to C–N stretching vibrations and amide III (around 1241 cm^−1^ and 1243 cm^−1^ in HCB-CS and HCR-CS complexes, respectively (C–N stretching and N–H bending vibrations of amide linkages) were similar to the specific absorption bands found in collagen [21,36]. The interaction between amino groups from chitosan and carboxyl groups of collagen led to the formation of H bonds [56]. The band intensity around 1640 cm^−1^ for lemon EO and dill EO could be observed for HCB-CS compositions. These bands are also evident in the spectra for HCB and HCR containing encapsulated EOs. The synergic effect between dill EO and lemon balm EO is observed in the FTIR spectra by decreasing the intensity of specific bands due to the interaction between the hydrophobic groups present in the collagen hydrolysate and chitosan and essential oils. Therefore, it is expected that the bioactive compounds of dill EO and lemon balm EO are present within the electrospun collagen-chitosan complex nanofibers.

### 3.5. Antimicrobial Activity

Data on the diameters of the inhibition zones (mm) are presented in Table 4 and Supplementary Materials Figures S1–S7.

Essential oil from dill (*Anethum graveolens* L.) seeds was slightly efficient only against *S. aureus* and *C. glabrata*, while the sample containing lemon balm EO had a very small antimicrobial activity against the same strains and also against *E. coli* and *E. faecalis*. The combination of dill EO with lemon balm EO reduced the antimicrobial activity of the previously tested samples.

The electrospun sample based on collagen hydrolysate extracted from bovine tendons mixed with chitosan (coded HCB) was found to be efficient against almost all the tested reference strains, excepting *E. faecalis*. It was observed that the antibacterial assays of HCB-CS nanofibers against *S. aureus* were lower than that of *E. coli,* due to the higher hydrophilicity of the Gram-negative bacteria as compared with the Gram-positive species, making them more susceptible to membrane degradation [57]. Instead, the electrospun sample based on collagen hydrolysate extracted from rabbit skin mixed with chitosan (coded HCR) was very efficient against all the tested samples, excepting *E. coli*. A high inhibition effect towards *S. aureus* was also reported in the case of collagen/chitosan scaffolds [58].

The combination of HCB-CS with dill EO or lemon balm EO increased the antimicrobial activity, and the combination with both essential oils sometimes increased the antimicrobial activity (against *S. aureus*, *E. faecalis*, *C. albicans*, and *C. glabrata*) and sometimes decreased the antimicrobial activity against *S. typhimurium* and *A. brasiliensis*.

The HCR-CS activity was also increased in the presence of dill EO or lemon balm EO, and the addition of both oils led to more efficient activity in the case of *S. aureus* or a less efficient activity against *E. faecalis* and *A. brasiliensis*. A similar increase in the antimicrobial activity of collagen nanofibers against *S. aureus*, *E. coli, P. aeruginosa,* and *C. albicans* [36] or chitosan-polyvinyl alcohol (PVA) film against *S.aureus* and *P. aeruginosa* [59] in the presence of EOs was reported.

For the other microorganism strain tests, the presence of the essential oils did not modify the antimicrobial activity for the HCR electrospun sample. These findings are related to the known antimicrobial activity of chitosan as well as to the encapsulated EOs for which the release from polymeric matrix was probably more difficult. Other authors have reported inhibition of *E. coli* in the case of cellulose acetate nanofibers loaded with EOs [9] due to the high exposed surface area of the fibers, as well as to the microorganism possibility to diffuse inside the network mats, favoring contact with the bioactive compounds of EOs. The different behaviors of the microorganism tests to the electrospun fibers could be explained due to the pores created in the fibrous network (according to Figure 2, when different dimensions can be observed). Depending on the microorganism test dimension, it was able to penetrate the network of the electrospun fibers. Thus, the size dimensions for *E. coli* cells and *C. albicans* cells are 1.5 and 4 µm, respectively [9].

### 3.6. In Vivo Biocompatibility Evaluation

Throughout the experiment, there were no changes in the behavior of the animals to which the studied electrospun samples were administered; they performed the specific movements of environmental exploration, feeding, watering, and personal hygiene.

On the seventh day of the experiment, the patches were removed, and the incision area was macroscopically evaluated. It was pointed out that, both in the animals from the control group as well as in those that received electrospun samples without and with encapsulated essential oils, the incision area was scarred and did not show the appearance of inflammation.

#### 3.6.1. Hematological Tests

The laboratory examination showed that the percentage values of the components in the leukocyte formula (neutrophil polymorphonuclear (PMN), lymphocytes (Ly), eosinophils (E), monocytes (M), and basophils (B)) in blood collected from animals that received electrospun nanofibers with and without essential oil, were comparable with those in the control group (coded C), both at 24 h and 7 days (Table 5).

#### 3.6.2. Activity of Liver Enzymes

Table 6 shows the glutamic-oxaloacetic transaminase (TGO), glutamic-pyruvic transaminase (TGP), and lactate dehydrogenase (LDH) serum values for mice that received electrospun samples.

According to Table 6, no significant variations in TGP, TGO, and LDH levels were observed in animals that received electrospun samples as compared with animals in the control group, at the two time points of the determination.

Table 7 shows the urea and creatinine values in mice that received electrospun samples.

The application of electrospun samples containing encapsulated essential oil, or not, did not produce substantial variations in serum levels of urea and creatinine as compared with the control group, after one day and 7 days, respectively, in the experiment.

Table 8 shows the superoxide dismutase (SOD) and glutathione peroxidase (GPx) values in the blood of mice that received electrospun samples.

#### 3.6.3. Immunological Tests

The application of electrospun samples was not followed by substantial changes in serum opsonic capacity (OC), phagocytic capacity (PC), and bactericidal capacity (BC) as compared with animals in the control group after 7 days (Table 9).

In our experimental conditions, the administration of electrospun samples containing essential oils does not cause obvious hematological, biochemical, and immunological changes and does not significantly influence the specific parameters of oxidative stress, as compared with the marker group. These tests suggest good in vivo biocompatibility after administration in mice of electrospun samples based on hydrolyzed collagen extracted from bovine tendons or rabbit skin mixed with chitosan and containing dill and/or lemon balm essential oils as encapsulated bioactive compounds.

## 4. Conclusions

Bioactive collagen hydrolysate-chitosan nanofibers with or without lemon balm (*Melissa officinalis* L.) and dill (*Anethum graveolens* L.) essential oils (EOs) were successfully prepared by electrospinning for new wound dressing preparation. Two kinds of collagen hydrolysates, from bovine tendons and rabbit skins, were used in combination with chitosan in view of essential oils encapsulation by electrospinning. The characterization of new composite nanofibers by SEM and ATR-FTIR showed that the thin nanofibers of 60–120 nm average size were fabricated and the interaction of amino and hydroxyl groups from chitosan with carboxylic groups from collagen was suggested by the absence of amide II (1545 cm^−1^) band associated with the secondary structure in chitosan from all nanospun nanofibers. The intensity of band around 1640 cm^−1^ for lemon EO and dill EO could be identified in essential oil-loaded collagen hydrolysate-chitosan nanofibers as well as the specific bands intensity decreasing as an effect of component interaction in the electrospinning process.

The antimicrobial activity of electrospun bioactive composites showed that the nanofibers based on bovine collagen hydrolysate with chitosan are efficient against *Staphylococcus aureus* ATCC25923, *Escherichia coli* ATCC25922, *Salmonella typhimurium* ATCC14028, *Candida albicans* ATCC10231, *Candida glabrata* ATCC90028, and *Aspergillus brasiliensis* ATCC9642. The antimicrobial activity efficiency increased for essential oil-loaded bovine collagen hydrolysate-chitosan nanofibers against *S. aureus*, *E. faecalis*, *C. albicans*, and *C. glabrata*. The electrospun nanofibers based on rabbit skin collagen hydrolysate-chitosan were very efficient against all tested strains, excepting *E. coli*. The antimicrobial efficiency increased for essential oil-loaded rabbit collagen hydrolysate-chitosan in the case of *S. aureus*.

The in vivo biocompatibility tests of wound patches based on new electrospun nanofibers was achieved on white Swiss mice by analyzing the hematological (components in the leukocyte formula), biochemical (TGO, TGP and LDH serum values, urea and creatinine, SOD, and GPx), and immunological (serum opsonic capacity, phagocytic capacity, and bactericidal capacity) and showed their good biocompatibility as compared with a reference. Given that these nanofibers have been proven to show good biocompatibility in vivo, we can appreciate that they could be suitable for biomedical applications, especially for wound healing.

## Figures and Tables

**Table 1:** Total phenolic content (TPC) and antioxidant characteristics evaluated for dill EO and lemon balm EO at a concentration of 60 mg mL^−1^ in ethanol.

EO Type	TPC (mg GAE/g Dry Substance)	DPPH (%)	ABTS (%)
Dill	0.48	13.21	36.10
Lemon balm	0.87	23.11	46.17

Other chemicals used were of analytical grade.

**Table 2:** Label and composition of nanofibers containing collagen, chitosan, and encapsulated essential oils prepared by electrospinning technique. HCB-CS is control nanofibers based on collagen hydrolysate from bovine tendons mixed with chitosan solution; HCB-CS/D is dill EO encapsulated into electrospun collagen hydrolysate from bovine tendons mixed with chitosan solution; HCB-CS/L is lemon balm EO encapsulated into electrospun collagen hydrolysate from bovine tendons mixed with chitosan solution; HCB-CS/D&L is a mixture of dill and lemon balm EOs encapsulated into electrospun collagen hydrolysate from bovine tendons mixed with chitosan solution; HCR-CS is control nanofibers based on collagen hydrolysate from rabbit skins mixed with chitosan solution; HCR-CS/D is dill EO encapsulated into electrospun collagen hydrolysate from rabbit skins mixed with chitosan solution; HCR-CS/L is lemon balm EO encapsulated into electrospun collagen hydrolysate from rabbit skins mixed with chitosan solution; HCR-CS/D&L is a mixture of dill and lemon balm EOs encapsulated into electrospun collagen hydrolysate from rabbit skins mixed with chitosan solution.

Code	HCB-CS	HCR-CS	Dill EO	Lemon balm EO
HCB-CS	X			
HCB-CS/D	X		X	
HCB-CS/L	X			X
HCB-CS/D&L	X		X	X
HCR-CS		X		
HCR-CS/D		X	X	
HCR-CS/L		X		X
HCR-CS/D&L		X	X	X

**Table 3:** Characteristics for hydrolyzed collagen from bovine tendons (HCB) and rabbit skins (HCR) in powder form [23].

Characteristics	Values ± SD		Methods
	HCB	HCR	
Volatile matters, %	10.67 ± 0.35	9.10 ± 0.35	SR EN ISO 4684:2006
Ash content, %	nd	1.61 ± 0.20	SR EN ISO 4047:2002
Total nitrogen, %	16.74 ± 0.35	17.32 ± 0.35	SR EN ISO 5397:1996
Protein, %	94.06 ± 0.35	97.28 ± 0.35	SR EN ISO 5397:1996
Aminic nitrogen, %	0.65 ± 0.24	0.87 ± 0.24	ICPI Method
Molecular weight, Da	22500	15000 ± 78	Sorensen Method
Conductivity (solution 10% in distilled water), μS/cm	0.57	820 ± 0.15	SR EN 2788:1997
pH (solution 10% in distilled water), pH units	4.40	7.50 ± 0.11	STAS 8619/3: 1990
Average particle size, nm		926.7	
Polydispersity		0.510	
Zeta potential		5.53	

**Table 4:** Inhibition zone (mm) of prepared electrospun samples in contact with different microorganism tests.

Sample	*S. aureus*	*E. coli*	*E. faecalis*	*S. typhimurium*	*C. albicans*	*C. glabrata*	*A. brasiliensis*
D	8.94 ± 0.04	-	-	-	-	16.34 ± 0.14	-
L	9.04 ± 0.25	9.09 ± 0.12	8.42 ± 0.14	-	-	12.24 ± 0.35	-
D&L	7.86 ± 0.45	8.12 ± 0.07	7.00 ± 0.31	-	-	8.06 ± 0.18	-
HCB-CS	12.94 ± 0.31	17.21 ± 0.04	-	17.47 ± 0.11	18.29 ± 0.28	22.50 ± 0.34	23.64 ± 0.27
HCB-CS/D	11.19 ± 0.18	19.09 ± 0.31	16.12 ± 0.08	15.33 ± 0.35	15.69 ± 0.07	26.53 ± 0.24	16.72 ± 0.47
HCB-CS/L	17.39 ± 0.21	25.09 ± 0.11	26.70 ± 0.12	18.87 ± 0.54	17.41 ± 0.31	22.50 ± 0.54	15.62 ± 0.32
HCB-CS/D&L	26.43 ± 0.05	22.79 ± 0.41	25.28 ± 0.51	13.19 ± 0.11	19.61 ± 0.23	30.35 ± 0.33	14.68 ± 0.22
HCR-CS	20.67 ± 0.21	-	28.56 ± 0.23	29.88 ± 0.27	19.05 ± 0.17	16.03 ± 0.47	20.03 ± 0.08
HCR-CS/D	21.40 ± 0.17	10.27 ± 0.12	26.79 ± 0.12	30.88 ± 0.13	19.60 ± 0.12	42.58 ± 0.57	16.14 ± 0.21
HCR-CS/L	34.93 ± 0.07	9.46 ± 0.13	28.71 ± 0.24	27.54 ± 0.24	19.47 ± 0.05	51.12 ± 0.24	10.74 ± 0.26
HCR-CS/D&L	35.46 ± 0.07	12.36 ± 0.21	24.72 ± 0.11	28.83 ± 0.17	18.84 ± 0.21	46.03 ± 0.07	11.78 ± 0.33

**Table 5:** Changes in the percentage values of the components in the leukocyte formula in animals that received electrospun samples. Values are expressed as arithmetic mean ± SD of the average percentage of components in the leukocyte formula for 5 mice per batch.

Sample	Period	Leukocyte Formula (%)				
		PMN	Ly	E	M	B
Control	24 h	28.3 ± 9.5	65.2 ± 19.1	0.1 ± 0.05	6.2 ± 1.3	0.2 ± 0.1
	7 days	28.6 ± 9.3	64.7 ± 18.7	0.1 ± 0.05	6.4 ± 1.1	0.2 ± 0.05
HCB-CS	24 h	27.8 ± 9.7	65.6 ± 18.9	0.1 ± 0.05	6.3 ± 1.1	0.2 ± 0.1
	7 days	28.6 ± 9.5	64.6 ± 19.5	0.2 ± 0.05	6.4 ± 1.5	0.2 ± 0.1
HCB-CS/D	24 h	27.6 ± 9.1	65.9 ± 19.3	0.2 ± 0.1	6.1 ± 1.3	0.2 ± 0.05
	7 days	28.5 ± 9.3	65.0 ± 17.9	0.1 ± 0.05	6.2 ± 1.1	0.2 ± 0.05
HCB-CS/L	24 h	28.3 ± 9.7	65.2 ± 19.1	0.1 ± 0.05	6.2 ± 1.3	0.2 ± 0.05
	7 days	28.8 ± 8.9	64.5 ± 18.5	0.2 ± 0.05	6.3 ± 1.3	0.2 ± 0.1
HCB-CS/D&L	24 h	28.4 ± 8.3	64.9 ± 19.7	0.2 ± 0.05	6.3 ± 1.1	0.2 ± 0.05
	7 days	28.7 ± 8.5	64.5 ± 19.3	0.2 ± 0.05	6.4 ± 1.5	0.2 ± 0.1
HCR-CS	24 h	28.3 ± 9.1	65.1 ± 19.5	0.2 ± 0.1	6.2 ± 1.1	0.2 ± 0.05
	7 days	28.5 ± 8.3	64.8 ± 19.3	0.2 ± 0.05	6.3 ± 1.1	0.2 ± 0.05
HCR-CS/D	24 h	28.6 ± 8.7	65.0 ± 19.1	0.1 ± 0.05	6.1 ± 1.3	0.2 ± 0.05
	7 days	28.5 ± 8.5	64.8 ± 18.7	0.1 ± 0.05	6.4 ± 1.5	0.2 ± 0.05
HCR-CS/L	24 h	27.8 ± 9.3	65.7 ± 18.5	0.1 ± 0.05	6.2 ± 1.3	0.2 ± 0.05
	7 days	28.3 ± 8.9	65.1 ± 19.3	0.2 ± 0.05	6.2 ± 1.3	0.2 ± 0.1
HCR-CS/D&L	24 h	27.6 ± 8.5	65.7 ± 19.5	0.2 ± 0.1	6.3 ± 1.1	0.2 ± 0.1
	7 days	28.7 ± 9.1	64.7 ± 19.1	0.1 ± 0.05	6.3 ± 1.3	0.2 ± 0.05

**Table 6:** Changes in glutamic-oxaloacetic transaminase (TGO), glutamic-pyruvic transaminase (TGP), and lactate dehydrogenase (LDH) serum values for animals that received electrospun samples. Values are expressed as arithmetic mean ± SD of the TGO, TGP, and LDH average values for 5 mice per batch.

Sample	Period	TGP (U/mL)	TGO (U/mL)	LDH (U/L)
Control	24 h	40.4 ± 11.5	163.4 ± 34.7	330.73 ± 72.45
	7 days	41.9 ± 12.1	167.7 ± 35.5	332.67 ± 77.37
HCB-CS	24 h	39.7 ± 12.3	164.5 ± 35.3	329.35 ± 69.73
	7 days	40.3 ± 11.3	168.3 ± 36.5	331.27 ± 74.65
HCB-CS/D	24 h	38.5 ± 11.5	166.7 ± 40.1	331.83 ± 78.33
	7 days	39.7 ± 11.7	169.5 ± 38.3	330.55 ± 80.29
HCB-CS/L	24 h	40.4 ± 11.3	165.3 ± 34.7	329.37 ± 76.55
	7 days	41.3 ± 12.1	170.3 ± 33.9	331.51 ± 75.17
HCB-CS/D&L	24 h	40.6 ± 11.7	168.7 ± 39.5	330.33 ± 79.39
	7 days	42.2 ± 11.5	171.1 ± 35.7	333.67 ± 80.55
HCR-CS	24 h	39.5 ± 12.1	165.7 ± 36.1	331.83 ± 79.67
	7 days	41.4 ± 11.7	164.3 ± 33.5	331.19 ± 75.29
HCR-CS/D	24 h	40.1 ± 11.5	166.9 ± 35.7	330.17 ± 74.73
	7 days	40.8 ± 11.9	166.7 ± 37.3	331.45 ± 81.35
HCR-CS/L	24 h	40.3 ± 11.3	165.9 ± 35.3	330.17 ± 77.65
	7 days	41.6 ± 11.7	165.7 ± 38.5	329.67 ± 79.83
HCR-CS/D&L	24 h	39.9 ± 12.1	167.7 ± 36.3	331.55 ± 76.19
	7 days	42.1 ± 11.5	169.3 ± 35.7	332.43 ± 74.37

**Table 7:** Changes in blood levels of urea and creatinine in animals that received electrospun samples. Values are expressed as arithmetic mean ± SD of the average of urea and creatinine levels for 5 mice per batch.

Sample	Test Period	Urea (mg/dL)	Creatinine (mg/dL)
Control	24 h	28.9 ± 5.5	0.8 ± 0.1
	7 days	29.1 ± 6.1	0.9 ± 0.05
HCB-CS	24 h	28.5 ± 6.3	0.9 ± 0.05
	7 days	28.7 ± 5.7	0.9 ± 0.1
HCB-CS/D	24 h	29.1 ± 7.1	0.9 ± 0.01
	7 days	30.3 ± 6.7	0.8 ± 0.05
HCB-CS/L	24 h	29.3 ± 6.5	0.8 ± 0.05
	7 days	29.7 ± 6.1	0.8 ± 0.05
HCB-CS/D&L	24 h	30.1 ± 5.9	0.9 ± 0.05
	7 days	30.5 ± 7.3	0.8 ± 0.01
HCR-CS	24 h	30.9 ± 5.5	0.9 ± 0.05
	7 days	29.7 ± 5.7	1.0 ± 0.05
HCR-CS/D	24 h	29.9 ± 5.3	0.8 ± 0.01
	7 days	30.3 ± 6.7	0.9 ± 0.05
HCR-CS/L	24 h	29.7 ± 6.3	0.9 ± 0.05
	7 days	29.9 ± 5.9	0.9 ± 0.05
HCR-CS/D&L	24 h	30.1 ± 5.7	0.8 ± 0.05
	7 days	30.5 ± 5.5	0.8 ± 0.01

**Table 8:** Changes in the values of superoxide dismutase (SOD) and glutathione peroxidase (GPx) in the blood of animals that received electrospun samples. Values are expressed as arithmetic mean ± SD of the mean SOD and GPx values for 5 mice per batch.

Sample	Test Period	SOD (U/mg Protein)	GPx (µm/mg Protein)
Control	24 h	104.7 ± 18.7	12.7 ± 1.4
	7 days	103.6 ± 18.9	12.4 ± 2.1
HCB-CS	24 h	104.4 ± 18.7	12.2 ± 1.3
	7 days	104.8 ± 19.3	12.2 ± 1.3
HCB-CS/D	24 h	103.9 ± 18.5	12.4 ± 1.4
	7 days	105.3 ± 20.1	12.1 ± 1.4
HCB-CS/L	24 h	104.3 ± 18.7	12.7 ± 1.4
	7 days	104.9 ± 19.5	12.4 ± 2.1
HCB-CS/D&L	24 h	103.6 ± 19.3	12.2 ± 1.3
	7 days	104.7 ± 18.7	12.2 ± 1.3
HCR-CS	24 h	105.2 ± 18.5	12.4 ± 1.4
	7 days	104.5 ± 18.3	12.1 ± 1.4
HCR-CS/D	24 h	103.7 ± 18.7	12.7 ± 1.4
	7 days	105.1 ± 20.1	12.4 ± 2.1
HCR-CS/L	24 h	104.3 ± 18.5	12.2± 1.3
	7 days	104.8 ± 19.5	12.2 ± 1.3
HCR-CS/D&L	24 h	104.5 ± 18.3	12.4 ± 1.4
	7 days	105.0 ± 18.2	12.1 ± 1.4

No substantial variations of SOD and GPx activity were found in the animals that received the tested electrospun samples as compared with the control, at the two time points in the experiment.

**Table 9:** Changes in OC, PC, and BC values in animals that received electrospun samples. Values are expressed as arithmetic mean ± SD of the average values of OC, PC, and BC, for 5 mice per group.

Sample	Test Period	OC (colonies/mL)	PC (colonies/mL)	BC (colonies/mL)
Control	7 days	783.37 ± 64.73	521.63 ± 38.51	717.83 ± 60.19
HCB-CS	7 days	787.63 ± 70.47	519.37 ± 35.29	720.67 ± 48.45
HCB-CS/D	7 days	796.55 ± 61.33	518.43 ± 34.45	719.55 ± 55.27
HCB-CS/L	7 days	801.43 ± 67.17	524.51 ± 37.13	718.45 ± 58.67
HCB-CS/D&L	7 days	797.81 ± 66.67	522.55 ± 39.55	716.83 ± 51.53
HCR-CS	7 days	788.37 ± 59.45	520.17 ± 35.67	720.45 ± 49.45
HCR-CS/D	7 days	784.63 ± 65.51	521.29 ± 36.63	718.67 ± 52.17
HCR-CS/L	7 days	791.29 ± 68.29	528.45 ± 40.55	717.17 ± 54.33
HCR-CS/D&L	7 days	795.55 ± 70.55	523.67 ± 37.33	723.63 ± 57.13

## Data Availability

All data supporting reported results are included in the article.

## Supplementary Materials

The following are available online at https://www.mdpi.com/article/10.3390/pharmaceutics13111939/s1, Figure S1: Photographs of the antibacterial activity of the tested samples against *S. aureus*; Figure S2: Photographs of the antibacterial activity of the tested samples against *E coli*; Figure S3: Photographs of the antibacterial activity of the tested samples against *E. faecalis*; Figure S4: Photographs of the antibacterial activity of the tested samples against *S. typhimurium*; Figure S5: Photographs of the antifungal activity of the tested samples against *C. albicans*; Figure S6: Photographs of the antifungal activity of the tested samples against *C. glabrata*; Figure S7: Photographs of the antifungal activity of the tested samples against *A. brasiliensis*.
