# Supplementary material for: Bioactive Collagen Hydrolysate-Chitosan/Essential Oil Electrospun Nanofibers Designed for Medical Wound Dressings
Source: Pharmaceutics. 2021 Nov 16;13(11):1939. doi: 10.3390/pharmaceutics13111939 (PMC8621651; doi:10.3390/pharmaceutics13111939)
Supplement: Supplementary file 1 [file pharmaceutics-13-01939-s001.zip › pharmaceutics-1447159-supplementary.pdf]

pharmaceutics

MDPI

Supplementary Materials: Bioactive Collagen Hydrolysate - Chitosan/Essential Oils Electrospun Nanofibers Designed for Medical Wound Dressings

Maria Râpă, Carmen Gaidau, Liliana Mititelu-Tartau, Mariana-Daniela Berechet, Andrei Constantin Berbecaru, Irina Rosca, Aurica P. Chiriac, Ecaterina Matei, Andra-Mihaela Predescu and Cristian Predescu

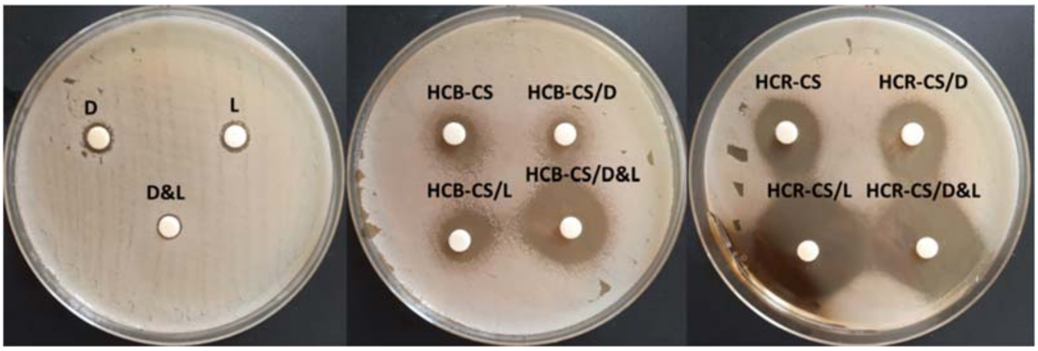

Figure S1. Photographs of the antibacterial activity of the tested samples against *S. aureus*.

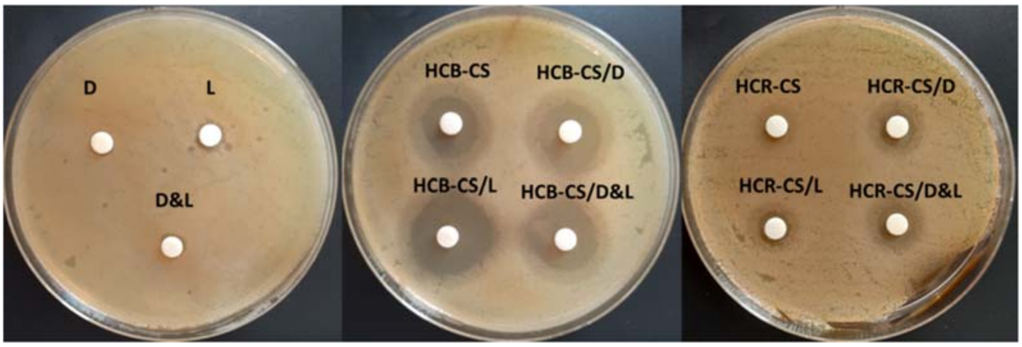

Figure S2. Photographs of the antibacterial activity of the tested samples against *E coli*.

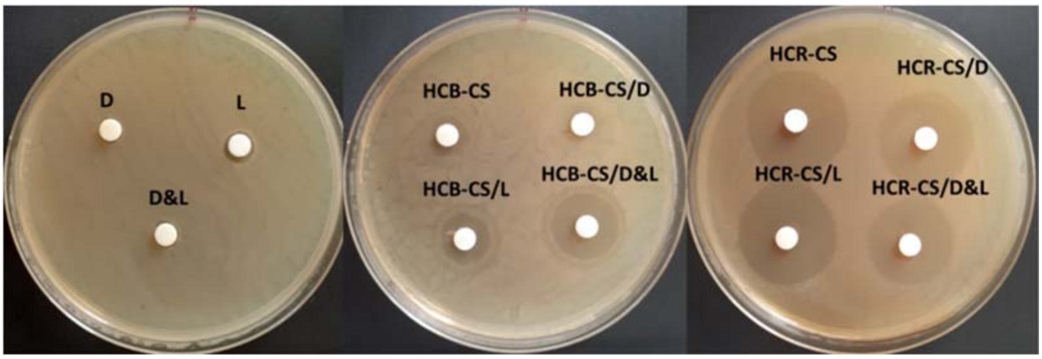

Figure S3. Photographs of the antibacterial activity of the tested samples against *E. faecalis*.

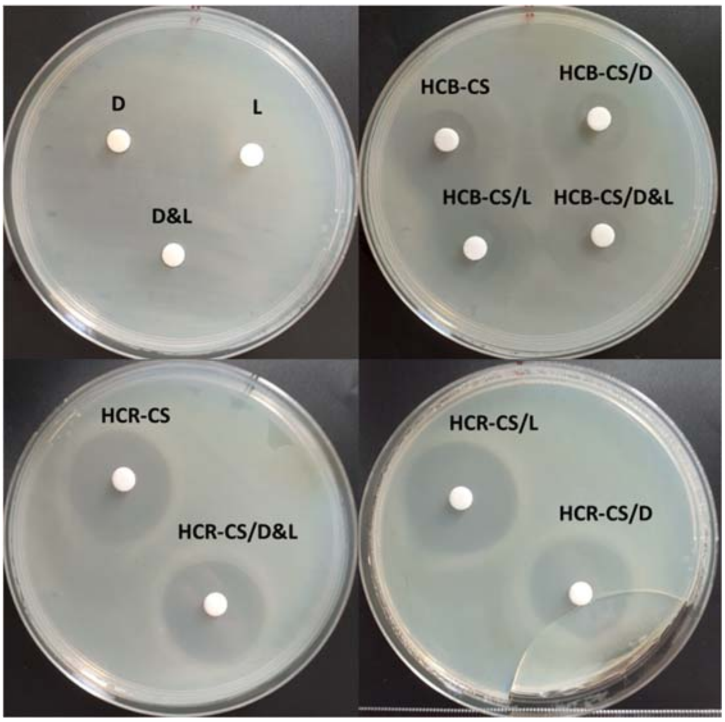

Figure S4. Photographs of the antibacterial activity of the tested samples against *S. typhimurium.*

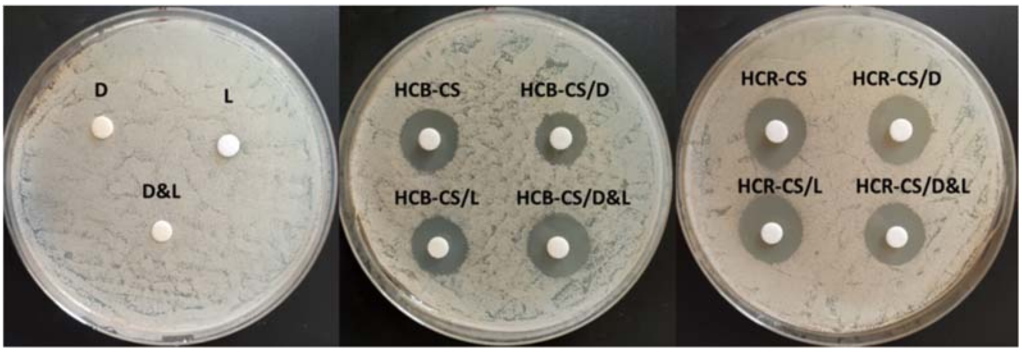

Figure S5. Photographs of the antifungal activity of the tested samples against *C. albicans*.

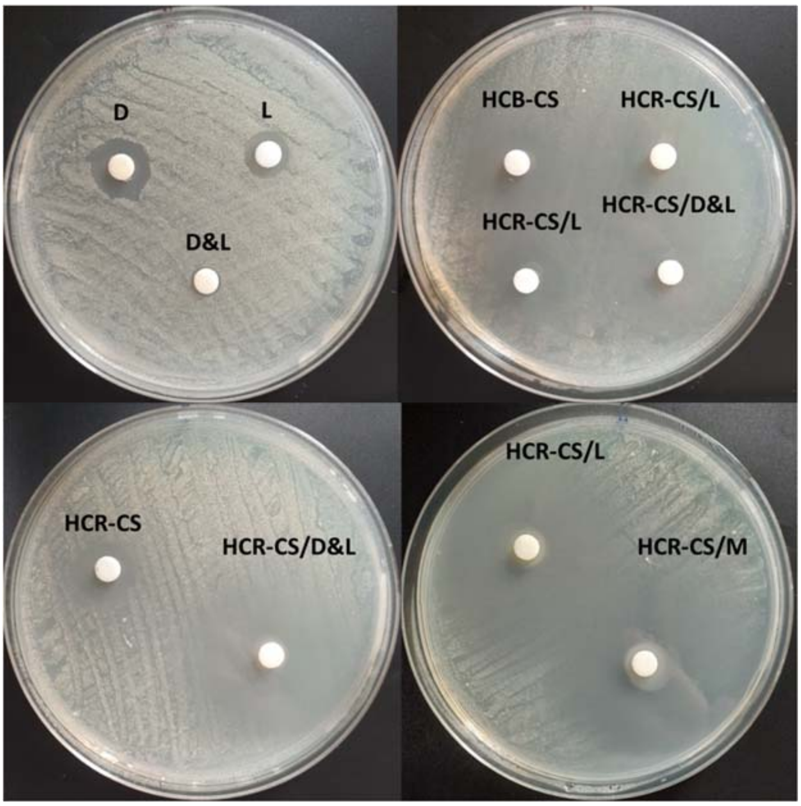

Figure S6. Photographs of the antifungal activity of the tested samples against *C. glabrata*.

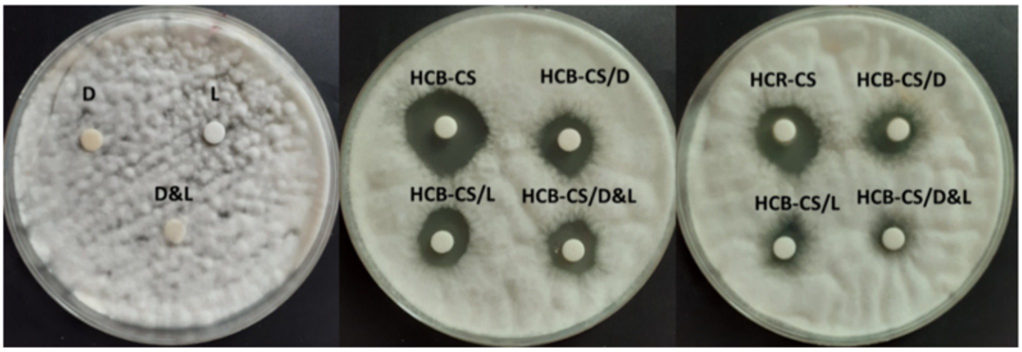

Figure S7. Photographs of the antifungal activity of the tested samples against *A. brasiliensis*.
